# Phosphane-Based Cyclodextrins as Mass Transfer Agents and Ligands for Aqueous Organometallic Catalysis

**DOI:** 10.3390/molecules171113062

**Published:** 2012-11-02

**Authors:** Sébastien Tilloy, Cécile Binkowski-Machut, Stéphane Menuel, Hervé Bricout, Eric Monflier

**Affiliations:** Unité de Catalyse et de Chimie du Solide (UCCS), CNRS, UMR 8181, Univ. Artois, Rue Jean Souvraz, SP 18, F-62307 Lens, France; Email: sebastien.tilloy@univ-artois.fr (S.T.); cecile.machut@univ-artois.fr (C.B.-M.); stephane.menuel@univ-artois.fr (S.M.); herve.bricout@univ-artois.fr (H.B.)

**Keywords:** cyclodextrin, hydroformylation, hydrogenation, inclusion, phosphanes, self-inclusion, water-soluble

## Abstract

The replacement of hazardous solvents and the utilization of catalytic processes are two key points of the green chemistry movement, so aqueous organometallic catalytic processes are of great interest in this context. Nevertheless, these processes require not only the use of water-soluble ligands such as phosphanes to solubilise the transition metals in water, but also the use of mass transfer agents to increase the solubility of organic substrates in water. In this context, phosphanes based on a cyclodextrin skeleton are an interesting alternative since these compounds can simultaneously act as mass transfer agents and as coordinating species towards transition metals. For twenty years, various cyclodextrin-functionalized phosphanes have been described in the literature. Nevertheless, while their coordinating properties towards transition metals and their catalytic properties were fully detailed, their mass transfer agent properties were much less discussed. As these mass transfer agent properties are directly linked to the availability of the cyclodextrin cavity, the aim of this review is to demonstrate that the nature of the reaction solvent and the nature of the linker between cyclodextrin and phosphorous moieties can deeply influence the recognition properties. In addition, the impact on the catalytic activity will be also discussed.

## 1. Introduction

**Figure 1:**
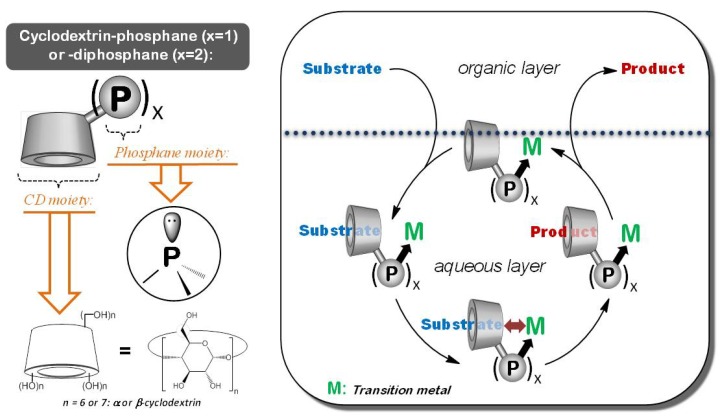
Principle of aqueous biphasic organometallic catalysis in the presence of cyclodextrin-functionalized phosphanes.

Green chemistry is a movement whose goal is to develop more environmentally friendly methods for the chemical industry and thus to reduce its environmental impact. Replacement of hazardous solvents with water is one of the main points of this movement [1]. In this field, aqueous organometallic catalysis occupies a prominent place [2]. Indeed, the use of organic solvents is not required and the water-soluble organometallic catalyst can be easily separated from products by decantation of the aqueous and organic phases at the end of the reaction. Nevertheless, aqueous organometallic catalysis processes present two main drawbacks. First, mass transfer agents, such as cyclodextrins (CDs) [3], are necessary to increase the low water-solubility of organic substrates and secondly, water-soluble ligands, such as phosphanes [4], are also necessary to solubilise the metal in water. In this context, cyclodextrin-functionalized phosphanes are an interesting alterative since these compounds can simultaneously act as mass transfer agents and as coordinating species (Figure 1). 

Indeed, CDs are water-soluble chemical receptors which are able to increase the water-solubility of organic substrates by inclusion into their hydrophobic cavity. In addition, the metal can be coordinated and rendered water-soluble thanks to the phosphane moiety covalently attached to the CD platform. For twenty years, various CD-mono, di, tri or tetra-phosphanes have been described in the literature [5,6,7,8,9,10,11,12,13,14,15,16,17,18,19,20,21,22,23,24,25,26,27], but only some CD-functionalized by one or two phosphorous moieties were exploited in aqueous organometallic catalytic processes [28,29,30,31,32,33,34,35,36]. Nevertheless, while their coordinating properties towards a transition metal and their catalytic properties were fully detailed, their mass transfer agent properties were much less discussed. As these mass transfer agent properties are directly linked to the recognition capacities, the aim of this review is to demonstrate that the nature of the reaction solvent and the nature of the linker between cyclodextrin and phosphorous moieties can deeply influence the availability of the CD cavity. In addition, the impact on the catalytic activity will be also discussed. 

## 2. Results and Discussion

### 2.1. Cyclodextrin-Diphosphanes

**Figure 2:**
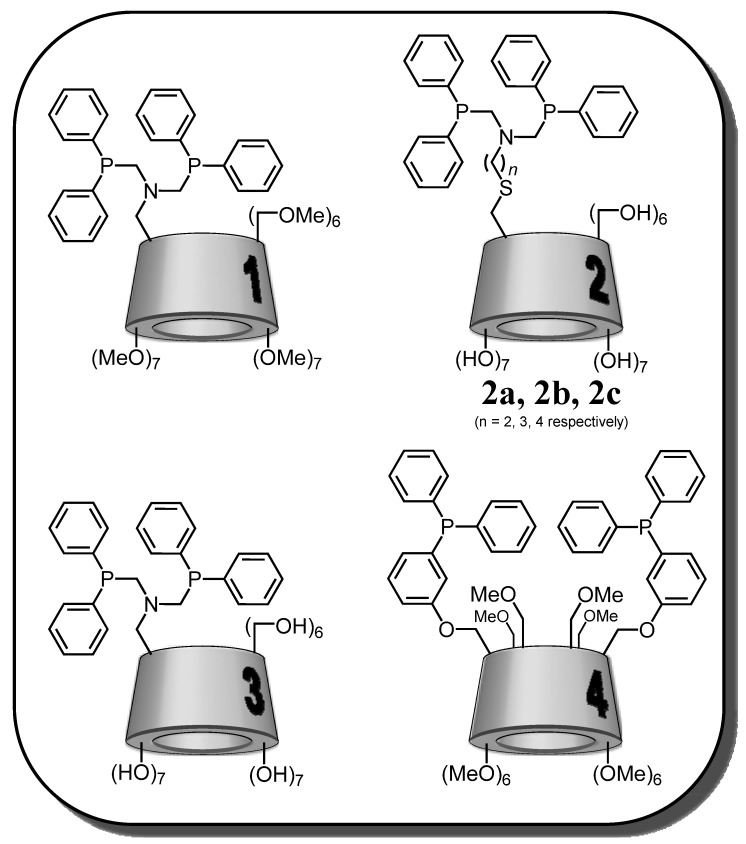
Schematic representation of cyclodextrin-diphosphanes.

Historically, the first studies were realized on the CD-diphosphanes series. The first example of phosphanes covalently bond to a CD and used during an aqueous organometallic process was described in 1997 by the Reetz group [36]. More precisely, this group has proposed three different types of CD-diphosphanes (Figure 2; CD-diphosphanes **1**–**3**) in order to combine phase transfer catalysis and transition metal catalysis. To establish whether these phosphane-CDs functioned as supramolecular catalysts, an equimolar mixture of 1-decene and 4-phenyl-1-butene were subjected to competition experiments. This mixture was hydrogenated in DMF by rhodium complexes based on these CD-diphosphanes. 

As a control experiment, a β-CD-free ligand was used instead of **1**, **2** or **3** and no substrate selectivity was achieved (Table 1, entry 1). In contrast, the use of CD-diphosphanes as ligands induced the preferential conversion of the phenyl-substituted alkene (Table 1, entries 2–5). This phenomenon was exacerbated when a DMF/H_2_O mixture was used as solvent (entries 6–8). The authors postulated that a recognition step precedes the hydrogenation and furthermore that the phenyl group preferentially enters into the hydrophobic cavity of the β-CD framework. This observation was supported by the fact that the substrate selectivity drastically decreases when working in the presence of *p*-xylene which competes with substrates for the space in the CD cavity (entry 9). Nevertheless, no spectroscopic proof to support this hypothesis was furnished.

**Table 1:** Substrate selectivity in hydrogenation.

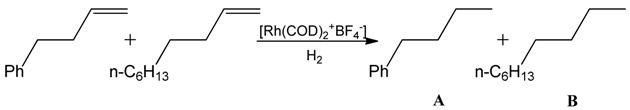			
**Entry**	**Ligand**	**Solvent**	**A/B**
1	PhN(CH_2_PPh_2_)_2_	DMF	50/50
2	****1****	DMF	68/32
3	****2a****	DMF	74/26
4	****2b****	DMF	71/29
5	****2c****	DMF	66/34
6	****1****	DMF/H_2_O (30/70)	82/18
7	****2a****	DMF/H_2_O (30/70)	81/19
8	****3****	DMF/H_2_O (30/70)	87/13
9 *	****3****	DMF/H_2_O (30/70)	57/43

* In the presence of *p*-xylene.

**Scheme 1:**
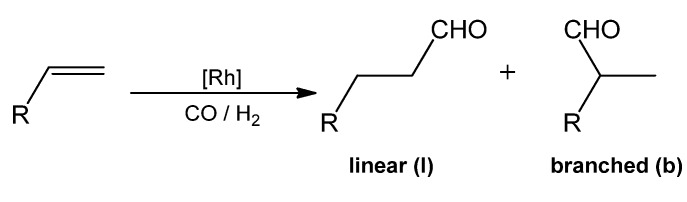
Rhodium-catalysed hydroformylation of terminal olefin.

**Figure 3:**
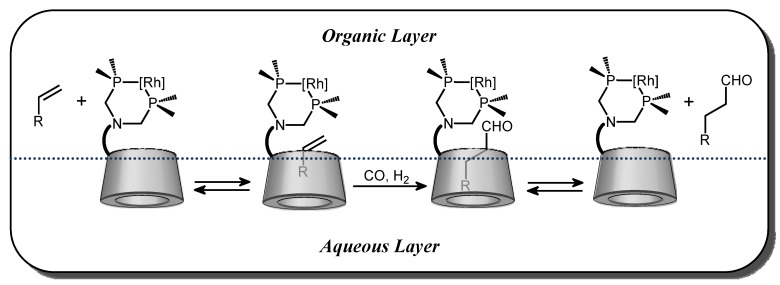
Mode of action of host-guest complexes in phase-transfer and rhodium catalysis proposed by Reetz.

Associated to a rhodium precursor, these β-CD-modified ligands were also tested in hydroformylation reactions in a two-phase system H_2_O (with 30% DMF)/olefin (Scheme 1). These complexes are able to perform hydroformylation of 1-octene, (*E*)-3-hexene and allylbenbenzene. In the case of 1-octene, unexpectedly high catalytic activities were observed with a chemoselectivity for aldehydes superior to 99%. These systems are 150 times more active at 80 °C than the traditional system Rh/TPPTS (TPPTS: tris(*m*-sulfonatophenyl)phosphane trisodium salt) at 120 °C. High regioselectivity was also observed. For instance, the change from Rh/PhN(CH_2_PPh_2_)_2_ in a one-phase system to Rh/**2a** in a two-phase system H_2_O (with 30% DMF)/olefin results in an increase in the linear/branched aldehydes ratio from 62/38 to 76/24. The authors explained this enhanced selectivity by the participation of a host-guest complex between the substrate and the CD cavity as exposed in the Figure 3. It is important to underline that in the presence of toluene, which also enters the CD cavity and thus plays the role of a competitor, the regioselectivity was reduced to 65/35. 

During the reduction reaction of nitro-halogenated aromatic derivatives in a biphasic mixture of water/toluene, **1** proved also to be very efficient when associated with Pt and Rh-precursors to produce the corresponding aromatic amines [33]. The authors postulated that the catalytic system (Pt/**1** or Rh/**1**) acts as a phase transfer catalyst probably on the basis of host/guest phenomena as already described above. Oddly, the use of toluene as organic layer was not discussed. Indeed, in the preceding hydroformylation work, it was stated that the presence of toluene modified the catalytic activity since it is a competitor for the substrate. 

The recovery and the recycling of the catalytic system during hydroformylation reactions were studied. The organic and aqueous phases were separated and unfortunately rhodium was detected by atomic absorption in the organic phase, showing a loss of the catalyst from the aqueous phase. Consequently, the reuse of the aqueous phase yielded a residual catalytic activity of approximately 50%. These last experiments showed that the ligands **1**–**3** were not able to effectively keep the rhodium in the aqueous phase. In addition, the presence of DMF which is used to facilitate the preparation of the catalyst, by increasing the solubility of the CD-phosphanes in water, also favoured the leaching of the metal into the organic phase. The presence of DMF has also a detrimental effect on the inclusion complexes. Indeed, DMF is a dissociating solvent which is well known to destabilize host-guest complexes [37]. Thus, in these preceding experiments, the presence of DMF disfavors not only the formation of stable inclusion complexes between the CD cavity and the substrate, but also leads to a loss of the catalyst in the organic layer.

Following these pioneering works, Matt and coworkers described the synthesis of a diphosphane based on permethylated α-cyclodextrin (Figure 2; CD-diphosphane **4**) [32]. This ligand is capable of complexing metals such as gold, silver, platinum, palladium and rhodium. The complex formed with rhodium has been tested in the hydroformylation reaction of 1-octene in biphasic aqueous medium (water/MeOH: 60/40). The conversion and chemoselectivity are greater than 99% and the l/b ratio is equal to 2.3. Unfortunately, as underlined by the authors, low water-solubility prevents “the catalyst” from acting as a supramolecular catalyst and leads to a significant loss of catalyst in the organic phase. Indeed, as in the case of DMF in the Reetz works, the presence of methanol, necessary as co-solvent to solubilize **4** in water, impedes the formation of stable inclusion complexes and leads to a loss of metal in the organic layer.

To summarize this part, these CD-diphosphanes **1**–**4** are poorly soluble in water forcing the use of a co-solvent with two major detrimental consequences: an increase in the loss of the catalyst in organic layer and a decrease in the CD/substrate recognition process. Since the presence of a co-solvent was necessary to increase the low water-solubility of these CD-diphosphanes leading to an adverse effect, CD-monophosphanes were synthetized. Indeed, the removal of one diphenylphosphino group was expected to reduce the compound hydrophobicity.

### 2.2. Cyclodextrin-Monophosphanes

The challenge was to synthetize CD-monophosphanes which are highly water-soluble and which possess highly recognition capacity in order to play both the role of mass transfer agent and coordinating species.

**Figure 4:**
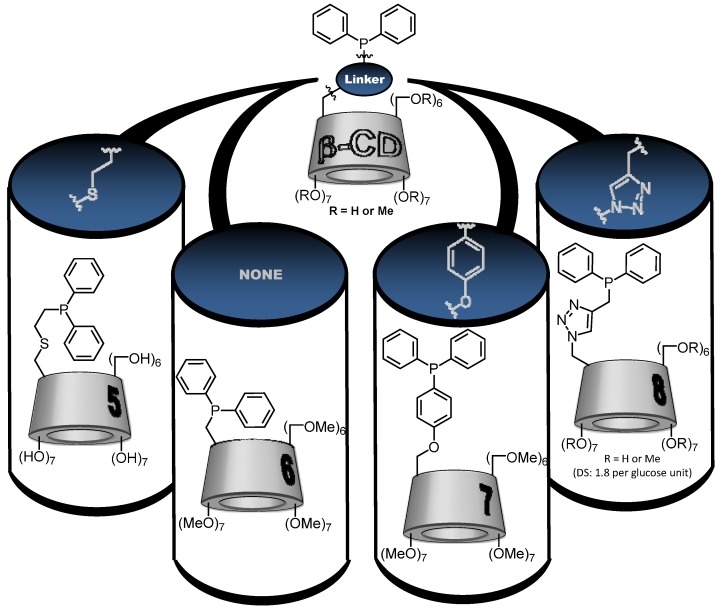
Schematic representations of cyclodextrin-monophosphanes.

**Figure 5:**
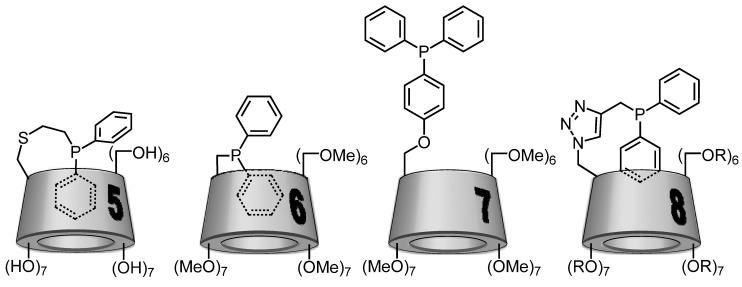
Schematic representation of preferential conformation of CD-phosphane **5**–**8**. For **8**, R= H or CH_3_ with a substitution degree of 1.8.

In 1998, Reetz and coworkers developed a monophosphane β-CD based on a native β-CD platform linked to a PPh_2_ group by a thioethylene unit (Figure 4; CD-phosphane **5**). There was no information on the water-solubility of **5**, but the self-inclusion of one phenyl group of the PPh_2_ moiety inside the CD cavity was shown by X-ray diffraction spectroscopy (Figure 5; CD-phosphane **5**) [38]. This ligand was evaluated in aqueous rhodium-catalyzed hydrogenation of olefins [34]. Unfortunately, hydrogenation occurred only under forcing conditions, leading to the formation of colloidal rhodium which then acted as a poor catalyst. 

In this context, between 2010 and 2012, our team has reported the synthesis and the catalytic applications of three new water-soluble CD-phosphanes. These CD-phosphanes were built on a per- or randomly-methylated β-CD platform attached to a diphenylphosphino group (PPh_2_). More precisely, the PPh_2_ group has replaced one OMe group of permethylated β-CD (Figure 4; CD phosphane **6**) [31] or was bound to the β-CD skeleton by two different kinds of linkers: a *p*-phenylenoxy (Figure 4; CD phosphane **7**) [30] or a 4-methylene-1,2,3-triazolyl group (Figure 4; CD phosphane **8**) [29].

The water-solubility of each CD-phosphane was equal to 3 mM, 0.01 mM and 15 mM at 20 °C for **6**–**8**, respectively. The difference between these values was firstly due to the nature of the CD platform. Indeed, the better water-solubility of **8** compared to **6** was due to the randomly methylated nature of its platform [39]. Secondly, the nature of the linker group between the CD and diphenylphosphino moieties directly impacted the positioning of the hydrophobic PPh_2_ group regarding the cavity and thus the water-solubility. In fact, as observed above in the case of the CD-phosphane **5**, intramolecular complexation can occur according to the nature of the linker.

For the three CD-phosphanes **6**, **7** or **8**, the potential self-inclusion of the diphenylphosphino group was studied by 2D T-ROESY NMR experiments. Based on these data, schematic representations were gathered in the Figure 5. For **6**, a real self-inclusion phenomenon is highlighted where one phenyl group is included in the CD cavity whereas for **8**, this behaviour is less marked and the primary face of the CD is only capped by one of the two phenyl groups. In the case of **7**, the diphenylphosphino group is outside the cavity.

As the availability of the CD cavity is primordial during a phase transfer catalysis process, some inclusion experiments were performed in the presence of an external guest. Sodium 1-adamantanecarboxylate (ACNa) was selected because of its high affinity for the β-CD cavity. Indeed, the value of association constant for the β-CD/ACNa complex was equal to 30,000 M^−1^ [40].

**Figure 6:**
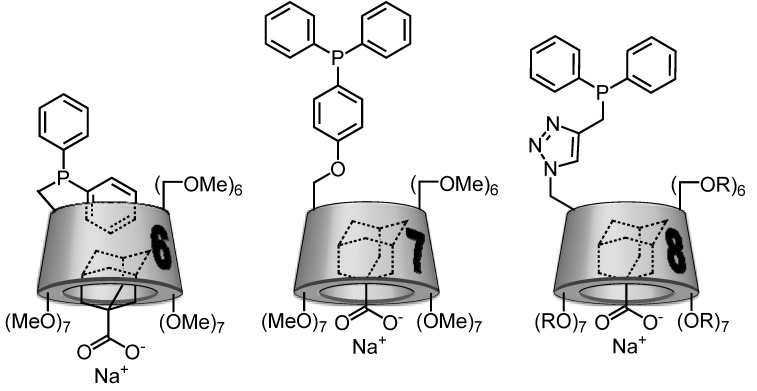
Schematic representation of the inclusion complexes between **6**, **7** or **8** and ACNa. For **8**, R = H or CH_3_ with a substitution degree of 1.8.

The study of the dipolar interactions through 2D T-ROESY NMR experiments performed on a mixture **7**/ACNa in D_2_O clearly revealed spatial proximity between the inner CD protons and guest protons proving the reality of an inclusion phenomenon. These observations show that **7** possess inclusion capacities. Attempts were also performed to include ACNa in the cavity of **6**. Interestingly, T-ROESY experiment showed dipolar contact not only between ACNa protons and CD protons, but also between phenyl protons and CD protons. Thus, the guest is not able to fully expel the self-included aromatic ring from the cavity. This phenomenon seems to prove that the inclusion capacity of **6** is low. This assertion was supported by the determination of the value of association constant with ACNa. This constant was found equal to 265 M^−1^. For comparison, the value of the association constant between ACNa and Trime-β-CD (permethylated β-CD) is equal to 10,700 M^−^^1^. This value, 40 times higher than in the case of **6**, signifies that the self-inclusion complex is very strongly stabilized and the occupation of the cavity of **6** by the aromatic moiety will prevent the formation of stable inclusion complexes. 2D T-ROESY NMR experiments performed on a solution containing **8** and ACNa showed correlation peaks between the adamantyl protons and internal CD protons. In addition, the correlation between the phenyl and internal CD protons observed in the case of **8** alone has disappeared. These observations unambiguously proved that ACNa is able to fully expulse the partially included phenyl group. The value of association constant for **8**/ACNa was found to be very high since equal to 46,600 M^−^^1^. This last result showed that although a capping phenomenon was observed, the CD cavity of **8** is able to deeply include a guest. Schematic representations of inclusion of ACNa in the cavity of **6**, **7** or **8** are summarized in Figure 6. 

All these preceding results showed that the water-solubility and the inclusion capacity of these CD-phosphanes depends on the nature of the linker group between the CD and diphenylphosphino moieties, so the influence of this linker was evaluated during catalytic experiments.

Associated to a rhodium precursor, the three CD-phosphanes **6**–**8** were efficient in aqueous hydrogenation of 2-methyl-3-buten-2-ol and in aqueous hydroformylation of 4-acetoxystyrene and methyl 4-pentenoate. These experiments showed that **6**–**8** were valuable ligands for the transformation of various water-soluble substrates [29,30,31]. 

The efficiency of these three CD-phosphanes as both ligands and mass transfer agent was explored in the case of 1-decene as an example of a water-insoluble substrate [41]. When rhodium complexes of CD-phosphanes **6** and **7** were tested in the hydroformylation reaction of 1-decene in biphasic aqueous medium, the conversion and chemoselectivity were greater than 97% with a l/b ratio equal to 2.8. Unfortunately, CD-phosphanes **6** or **7** are partially soluble in organic solvents. For example, the partition coefficient of **6** between water and heptane is equal to 0.48, that is, 68% of **6** stays in water and 32% moves to the organic layer. This behaviour is not in accordance with the specifications required for an application in biphasic aqueous organometallic catalytic processes. Indeed, a loss of the catalyst was observed in the organic layer thus impeding the recycling. When CD-phosphane **8** was used as ligand, the conversion did not exceed 5% after 6 h of reaction. This low conversion suggested that this ligand allowed immobilizing the rhodium in the aqueous layer. Unfortunately, it seems that 1-decene was not able to inverse the capping phenomenon by expulsing the phenyl group of the CD cavity. This behaviour prevents the transport of the olefin into the aqueous layer. To summarize this second part, the described CD-monophosphanes are not able to efficiently play both the role of mass transfer agent and catalytic species because of partial solubility in organic solvent or self-inclusion phenomena.

## 3. Conclusions

In this paper, we showed that it is not easy to synthetize CD-phosphanes able to act both as ligands and as phase transfer catalysts. Indeed, these CD-phosphanes have to be highly water-soluble to maintain the catalyst in aqueous layer and their hydrophobic cavity has to be available to include the substrate. The ideal CD-phosphane is expected to coordinate the metal center and to simultaneously form an inclusion complex with the substrate in order to achieve specific catalytic activity and selectivity. Up until 2012, no described CD-phosphane fulfills these specifications and the design of this ideal CD-phosphane remains a great challenge.

## References

[1] Anastas P.T., Warner J.C. (1998). Green Chemistry: Theory and Practice.

[2] Cornils B., Herrmann W.A. , Cornils B., Herrmann W.A. (2004). Aqueous-Phase Organometallic Catalysis.

[3] Hapiot F., Ponchel A., Tilloy S., Monflier E. (2011). Cyclodextrins and their applications in aqueous-phase metal-catalyzed reactions. C.R. Chim..

[4] Shaughnessy K.H. (2009). Hydrophilic ligands and their application in aqueous-phase metal-catalyzed reactions. Chem. Rev..

[5] Gramage-Doria R., Rodriguez-Lucena D., Armspach D., Egloff C., Jouffroy M., Matt D., Toupet L. (2011). A cavity-shaped diphosphane displaying oschelating behaviour. Angew. Chem. Int. Ed..

[6] Zaborova E., Deschamp J., Guieu S., Blériot Y., Poli G., Ménand M., Madec D., Prestat G., Sollogoub M. (2011). Cavitand supported tetraphosphine: Cyclodextrin offers a useful platform for Suzuki-Miyaura cross-coupling. Chem. Commun..

[7] Gramage-Doria R., Rodriguez-Lucena D., Armspach D., Egloff C., Jouffroy M., Matt D., Toupet L. (2011). Regioselective double capping of cyclodextrin scaffolds. Chem. Eur. J..

[8] Guieu S., Zaborova E., Blériot Y., Poli G., Jutand A., Madec D., Prestat G., Sollogoub M. (2010). Can hetero-polysubstituted cyclodextrins be considered as inherently chiral concave molecules?. Angew. Chem. Int. Ed..

[9] Armspach D., Matt D., Toupet L. (2009). Self-mediated, stereoselective oxidation of thia-capped cyclodextrins. Angew. Chem. Int. Ed..

[10] Poorters L., Armspach D., Matt D., Toupet L., Jones P.G. (2007). A metallocavitand functioning as a container for anions: formation of noncovalent linear assemblies mediated by a cyclodextrin-entrapped NO_3_−ion. Angew. Chem. Int. Ed..

[11] Poorters L., Armspach D., Matt D., Toupet L., Choua S., Turek P. (2007). Synthesis and properties of TRANSDIP, A rigid chelator built upon a cyclodextrin cavity: Is TRANSDIP an authentic trans-spanning ligand?. Chem. Eur. J..

[12] Poorters L., Armspach D., Matt D., Toupet L. (2007). α-TEPHOS: A cyclodextrin-derived tetraphosphine for multiple metal binding. Dalton Trans..

[13] Hapiot F., Tilloy S., Monflier E. (2006). Cyclodextrins as Supramolecular Hosts for Organometallic Complexes. Chem. Rev..

[14] Engeldinger E., Poorters L., Armspach D., Matt D., Toupet L. (2004). Diastereospecific synthesis of phosphinidene-capped cyclodextrins leading to “introverted” ligands. Chem. Commun..

[15] Engeldinger E., Armspach D., Matt D., Jones P.G. (2003). Cyclodextrin phosphanes as first and second coordination Sphere Cavitands. Chem. Eur. J..

[16] Poorters L., Armspach D., Matt D. (2003). Selective tetrafunctionalisation of alpha-cyclodextrin using the supertrityl protecting group. Synthesis of the first C2-symmetric tetraphosphine based on a cavitand (alpha-TEPHOS). Eur. J. Org. Chem..

[17] Engeldinger E., Armspach D., Matt D. (2003). Capped cyclodextrins. Chem. Rev..

[18] Engeldinger E., Armspach D., Matt D., Toupet L., Wesolek M.  (2002). . Synthesis of large chelate rings with diphosphites built on a cyclodextrin scaffold. Unexpected formation of 1,2-phenylene-capped α-cyclodextrins. C.R. Chim..

[19] Reetz M.T., Kostas I.D., Waldvogel S.R. (2002). Synthesis of a gold(I) complex with a (thio)phosphine-modified β-cyclodextrin. Inorg. Chem. Commun..

[20] Engeldinger E., Armspach D., Matt D., Jones P.G., Welter R. (2002). A cyclodextrin diphosphane as a first and second coordination sphere cavitand: evidence for weak C-H⋅⋅⋅Cl-M hydrogen bonds within metal-capped cavities. Angew. Chem. Int. Ed..

[21] Wong Y.T., Yang C., Ying K.C., Jia G. (2002). Synthesis of a novel beta-cyclodextrin-functionalized diphosphine ligand and its catalytic properties for asymmetric hydrogenation. Organometallics.

[22] Yang C., Cheung Y.K., Yao J., Wong Y.T., Jia G. (2001). Palladium and platinum complexes with a beta-cyclodextrin-functionalized phosphine ligand. Organometallics.

[23] Yang C., Wong Y.T., Li Z., Krepinsky J.J., Jia G. (2001). Synthesis of beta-cyclodextrin-functionalized (2S,4S)-(-)-4-(diphenylphosphino)-2-(diphenylphosphinomethyl) ligands and their rhodium and platinum complexes. Organometallics.

[24] Engeldinger E., Armspach D., Matt D. (2001). Cyclodextrin cavities as probes for ligand-exchange processes. Angew. Chem. Int. Ed..

[25] Deshpande R.M., Fukuoka A., Ichikawa M. (1999). Novel phosphinite capped cyclodextrin-rhodium catalysts in substrate selective hydroformylation. Chem. Lett..

[26] Sawamura M., Kitayama K., Ito Y.  (1993). Synthesis and properties of a new chiral diphosphine ligand bearing a cyclodextrin-based molecular recognition site and its palladium(II) complex. Tetrahedron: Asymmetry.

[27] Reetz M.T., Rudolph J.  (1993). Synthesis of a phosphine-modified cyclodextrin and its rhodium complex. Tetrahedron: Asymmetry.

[28] Hapiot F., Bricout H., Tilloy S., Monflier E. (2012). Functionalized Cyclodextrins as First and Second Coordination Sphere Ligands for Aqueous Organometallic Catalysis. Eur. J. Inorg. Chem..

[29] Tran D.N., Legrand F.X., Menuel S., Bricout H., Tilloy S., Monflier E. (2012). Cyclodextrin-phosphane possessing a guest-tunable conformation for aqueous rhodium-catalyzed hydroformylation. Chem. Commun..

[30] Legrand F.X., Six N., Slomianny C., Bricout H., Tilloy S., Monflier E. (2011). Synthesis, Rhodium complexes and catalytic applications of a new water-soluble triphenylphosphane-modified β-cyclodextrin. Adv. Synth. Catal..

[31] Machut-Binkowski C., Legrand F.X., Azaroual N., Tilloy S., Monflier E. (2010). New phosphane based on a β-cyclodextrin exhibiting a solvent-tunable conformation and its catalytic properties. Chem. Eur. J..

[32] Armspach D., Matt D. (1999). Metal-capped β-cyclodextrins: The crowing of the oligosaccharide torus with precious metals. Chem. Commun..

[33] Reetz M.T., Frömbgen C. (1999). Chemoselective reduction of halo-nitro aromatic compounds by β-cyclodextrin-modified transition metal catalysts in a biphasic system. Synthesis.

[34] Reetz M.T. (1998). Supramolecular transition metal catalysts in two-phase systems. Catal. Today.

[35] Reetz M.T. (1998). New supramolecular transition metal catalysis. J. Heterocycl. Chem..

[36] Reetz M.T., Waldvogel S.R. (1997). Diphosphanes as ligands for supramolecular rhodium catalysts. Angew. Chem. Int. Ed. Engl..

[37] Feliciano C.E. Quiñones (1999). The association of 4-(*N*,*N*-dimethylamino)benzonitrile and beta-cyclodextrin in dimethyl sulfoxide and N,N-dimethylformamide. J. Photochem. Photobiol. A Chem..

[38] Reetz M.T., Rudolph J., Goddard R. (2001). Diastereotopic group recognition in the solid state- A unique intramolecular β-cyclodextrin inclusion complex. Can. J. Chem..

[39] Loftsson T., Jahro P., Masson M., Jarvinen T.  (2005). Cyclodextrins in drug delivery. Expert Opin. Drug Deliv..

[40] Rekharsky M.V., Inoue Y. (1998). Complexation thermodynamics of cyclodextrins. Chem. Rev..

[41] Legrand F.X.  (2010). New mass transfer agents and ligands based on cyclodextrin for aqueous organometallic catalysis. Ph.D. Thesis.

